# Supplementary material for: Administration of nucleoside-modified mRNA encoding broadly neutralizing antibody protects humanized mice from HIV-1 challenge
Source: Nat Commun. 2017 Mar 2;8:14630. doi: 10.1038/ncomms14630 (PMC5337964; doi:10.1038/ncomms14630)
Supplement: Supplementary Information — Supplementary Figures [file ncomms14630-s1.pdf]

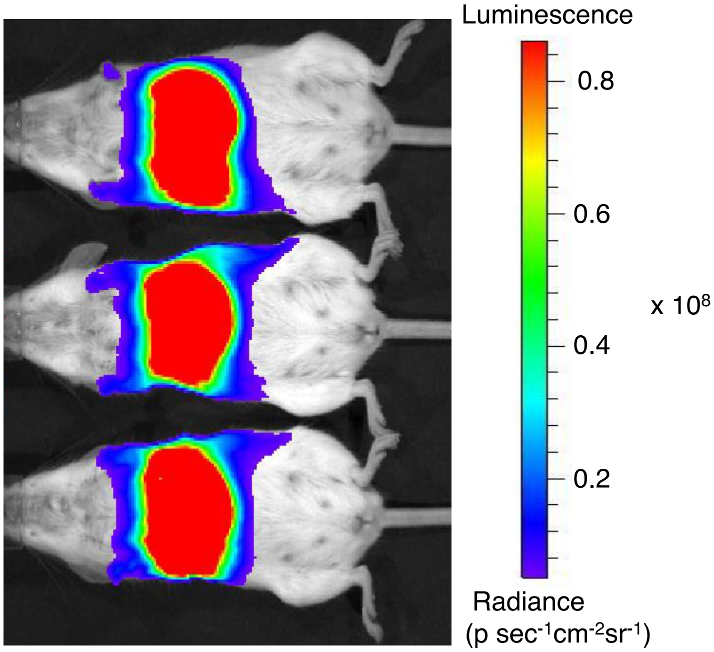

Supplementary Figure 1 Intravenously administered mRNA-LNPs target the liver. Representative IVIS image (24 hours post-injection) of BALB/c mice injected with 5 µg LNP-complexed firefly luciferase-encoding mRNA by the intravenous route.

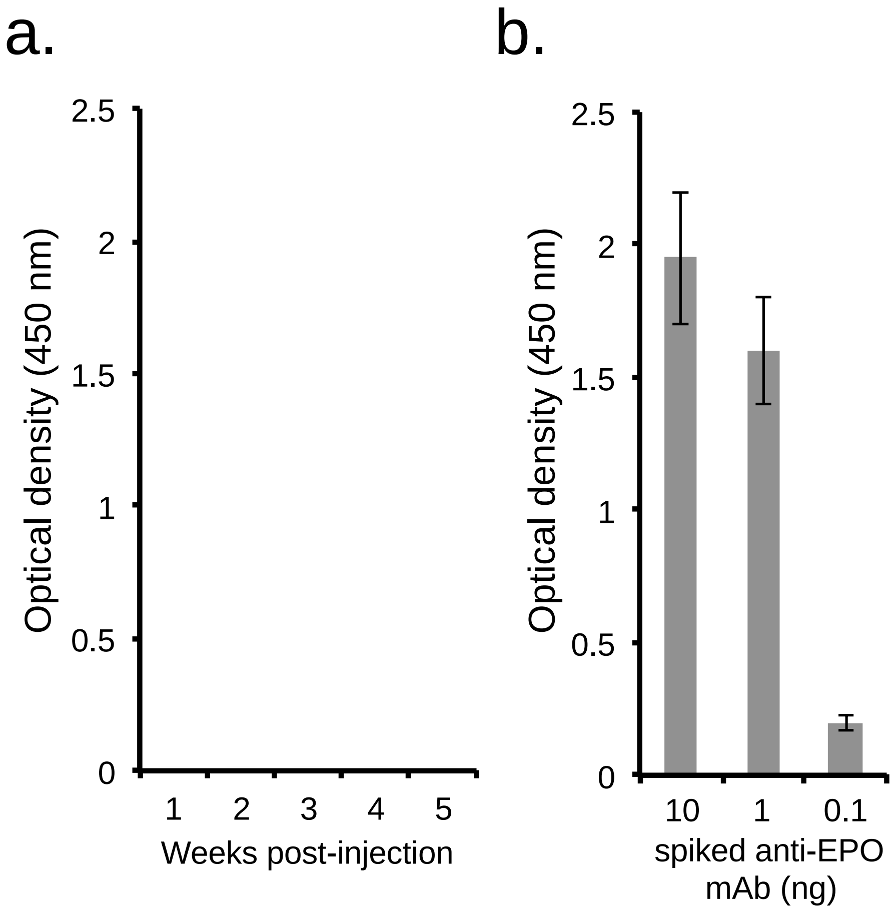

Supplementary Figure 2 Repeated delivery of nucleoside-modified mRNA into immunocompetent mice. BALB/c mice were intraperitoneally injected weekly for 5 weeks with 0.1 µg of murine erythropoietin (muEPO) encoding nucleoside-modified mRNA. **(a)** Animals were bled 1 week after each injection and antibody responses to muEPO protein were measured in the plasma by ELISA. **(b)** To measure the sensitivity of the ELISA, recombinant anti-muEPO antibodies (10, 1 and 0.1 ng) were spiked into mouse plasma and analyzed. Group size is 5 animals.

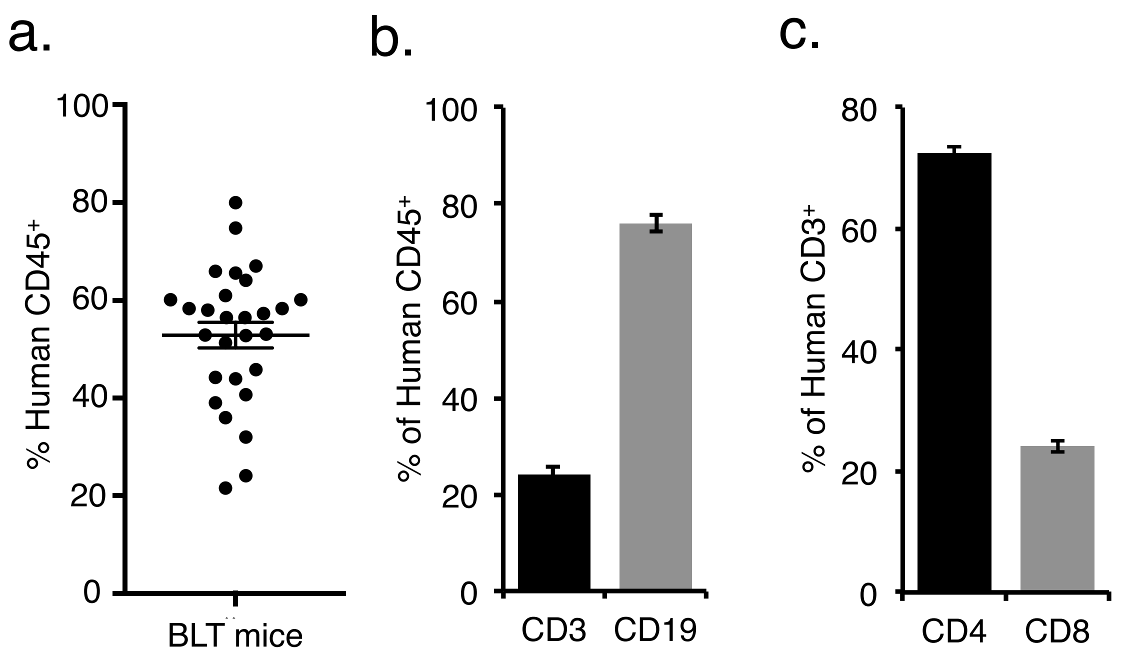

Supplementary Figure 3 Characterization of BLT humanized mice. BLT humanized mice were engrafted with 100,000 CD34$^+$ human hematopoietic stem cells. **(a)** Chimerism was measured in peripherial blood 16 weeks later by enumerating human CD45$^+$ cells in blood. **(b)** Percent of T cells (CD3$^+$) and B cells (CD19$^+$) of CD45$^+$ cells in BLT mice. **(c)** Percent of CD4$^+$ and CD8$^+$ T cells of total CD3$^+$ T cells in BLT mice. Group size is 28 animals.

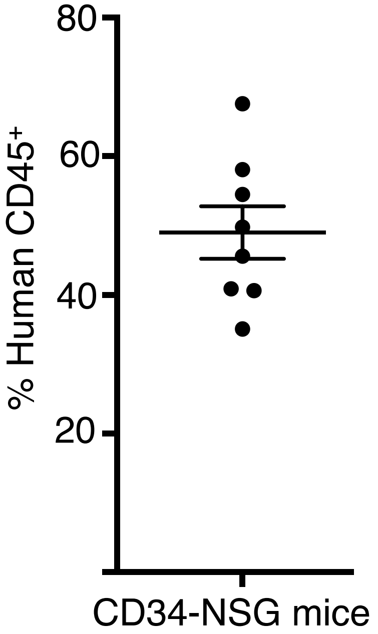

Supplementary Figure 4 Characterization of humanized CD34-NSG mice. NSG humanized mice were engrafted with 100,000 CD34^{+} human hematopoietic stem cells. Chimerism was measured in peripherial blood 12 weeks later by enumerating human CD45^{+} cells in blood. Group size is 8 animals.
